# Botulism From Drinking Prison-Made Illicit Alcohol — Arizona, 2012

**Published:** 2013-02-08

**Authors:** Graham Briggs, Shoana Anderson, Kenneth Komatsu, Joli Weiss, Evan Henke, Clarisse A. Tsang, Muhammad Vasiq, Agam K. Rao, Rajal K. Mody, Carolina Luquez, Janet K. Dykes, Jamae F. Morris, Tara C. Anderson, Laura E. Adams, Seema Yasmin

**Affiliations:** Pinal County Dept of Health; Arizona Dept of Health Svcs; Mountain Vista Medical Center, Mesa, Arizona; Div of Foodborne, Waterborne, and Environmental Diseases, National Center for Emerging Infectious and Zoonotic Disease; EIS officers, CDC

During November 24–27, 2012, the Arizona Department of Health Services (ADHS) was notified that eight male inmates of prison A, a maximum security prison, had been hospitalized for treatment of an acute neurologic condition suspected to be botulism. Botulism is a serious paralytic illness caused by a nerve toxin produced by the bacterium *Clostridium botulinum.* All eight patients reported drinking pruno, an illicitly brewed alcoholic beverage that has been associated with botulism outbreaks in prisons (1,2). This was the second outbreak of botulism in prison A during 2012; in August, four inmates were hospitalized for botulism after drinking pruno. Pinal County Health Services (PCHS), ADHS, and CDC investigated to identify the outbreak source, learn about pruno production, and provide recommendations for preventing future outbreaks of botulism in prisons.

A case of botulism was defined as signs and symptoms of cranial nerve palsies (e.g., double vision or blurred vision) and weakness, dysphagia, or impaired gag reflex, with onset in November 2012, in a prison A inmate with *Clostridium botulinum* bacteria or toxin in a clinical specimen or with a history of drinking pruno from the same batch as an inmate with a positive clinical specimen. The illnesses of eight male inmates aged 20–35 years met the case definition. The inmates were housed in two adjoining pods. All eight reported consuming pruno from a single batch on November 23, and had symptom onset November 24–26. All were hospitalized and received heptavalent botulinum antitoxin. Serum samples from all eight patients tested positive for botulinum toxin type A using mass spectrometry and mouse bioassay. Because of respiratory muscle paralysis, seven patients were intubated and were fed through percutaneous endoscopic gastrostomies. The seven were intubated for a range of 11–14 days before receiving tracheostomies.

An investigation by PCHS, ADHS, and CDC identified a batch of pruno as the outbreak source. This batch tested positive for botulinum toxin type A. Pruno typically is made by fermenting fruit and sugar in water; other commonly used ingredients include potatoes, corn, bread, and rice. Both prison A outbreaks were associated with pruno made with potatoes, as were outbreaks at prisons in California and Utah that have been reported since 2004 (Table) (1,2).

In 2004, four inmates of a California prison were hospitalized with pruno-related botulism; two patients required intubation. In 2005, one inmate of a California prison was hospitalized with botulism and intubated (2). An outbreak of botulism related to pruno occurred in a Utah maximum security prison in 2011 when eight inmates were hospitalized, and three of those patients were intubated (1). During a previous outbreak of botulism in prison A in August 2012, four different inmates were hospitalized, and one of those patients was intubated. Measures to prevent botulism in prison A were not instituted by prison authorities following the August outbreak. Since the recent outbreak of botulism, prison A has banned potatoes from the prison kitchen. Discussions are under way to ban sugar and other ingredients commonly used to make pruno that are available on the menu and in the prison store.

To prevent future outbreaks of botulism in prisons, ADHS and PCHS are assessing inmates’ knowledge of pruno production and risks associated with drinking pruno. Findings from this investigation will be used to plan inmate and prison staff education programs.

## Figures and Tables

**TABLE:** Characteristics of previously reported outbreaks of botulism associated with drinking prison-made illicit alcohol — United States, 2004–2012

Year	State	No. of cases	Age range (yrs)	No. hospitalized*	No. intubated
2004	California	4	19–35	4	2
2005	California	1	30	1	1
2011	Utah	8	24–35	8	3
2012	Arizona	4	27–33	4	1
2012	Arizona	8	20–35	8	7

* No deaths were reported.
